# Crystallization and preliminary structural analysis of the giant haemoglobin from *Glossoscolex paulistus* at 3.2 Å

**DOI:** 10.1107/S090904951002772X

**Published:** 2010-11-05

**Authors:** J. F. R. Bachega, L. Bleicher, E. R. Horjales, P. S. Santiago, R. C. Garratt, M. Tabak

**Affiliations:** a Centro de Biotecnologia Molecular Estrutural, Instituto de Fisica de São Carlos, Universidade de São Paulo, São Carlos – SP, CEP 13566-590, Brazil; b Instituto de Química de São Carlos, Universidade de São Paulo, São Carlos – SP, CEP 13566-590, Brazil

**Keywords:** haemoglobin, erythrocruorin, crystallization, earthworm

## Abstract

*Glossoscolex paulistus* is a free-living earthworm encountered in south-east Brazil. Its oxygen transport requirements are undertaken by a giant extracellular haemoglobin, or erythrocruorin (HbGp), which has an approximate molecular mass of 3.6 MDa and, by analogy with its homologue from *Lumbricus terrestris* (HbLt), is believed to be composed of a total of 180 polypeptide chains. In the present work the full 3.6 MDa particle in its cyanomet state was purified and crystallized using sodium citrate or PEG8000 as precipitant. The crystals contain one-quarter of the full particle in the asymmetric unit of the *I*222 cell and have parameters of *a* = 270.8 Å, *b* = 320.3 Å and *c* = 332.4 Å. Diffraction data were collected to 3.15 Å using synchrotron radiation on beamline X29A at the Brookhaven National Laboratory and represent the highest resolution data described to date for similar erythrocruorins. The structure was solved by molecular replacement using a search model corresponding to one-twelfth of its homologue from HbLt. This revealed that HbGp belongs to the type I class of erythrocruorins and provided an interpretable initial electron density map in which many features including the haem groups and disulfide bonds could be identified.

## Abstract 2

Diffraction data to 3.2 Å from crystals of the 3.6 MDa erythrocruorin from a Brazilian earthworm represent the highest resolution reported to date for similar complexes. An unambiguous molecular replacement solution shows the particle to belong to the type I class.

## 1. Introduction

The annelid erythrocruorins are giant hexagonal bilayer haemoglobins with molecular masses within the megadalton range. They are multimeric assemblies built from the association of both globin and non-globin chains which typically present a highly cooperative behaviour (Weber & Vinogradov, 2001 ▶). The crystal structures of two types of such erythrocruorins have thus far been described, those from *Lumbricus terrestris* (HbLt) (Royer *et al.*, 2006 ▶) and *Arenicola marina* (HbAc) (Royer *et al.*, 2007 ▶). In both cases the architecture of the full particle of approximately 3.6 MDa is based on two hexagonal discs in which the most prominent substructure corresponds to one-twelfth of the particle (a protomer) and is composed of a dodecamer of globins together with three non-globin chains called linkers. Both structures possess 622 (or *D*_6_) symmetry but present slightly different relative orientations of the two discs. In type I structures, such as that from HbLt, the hexagonal layers are staggered with respect to one another by approximately 16°, while in type II structures, such as that seen in HbAc, the two layers are eclipsed. These arrangements as seen in the crystal structures are consistent with those observed in three-dimensional reconstructions based on cryo-electron microscopy images (Jouan *et al.*, 2001 ▶) and would therefore appear to be intrinsic structural differences inherent to the two classes of molecule rather than alternative quarternary states accessible to a single species. Besides earthworms, cryo-electron microscopy has also established that the type I architecture is also observed in both leeches (de Haas, Biosset *et al.*, 1996 ▶) and a hydrothermal vent tube worm (de Haas, Zal *et al.*, 1996 ▶), whereas the type II class is only well established for polychaetes (de Haas, Taveau *et al.*, 1996 ▶).

A full particle of HbLt is composed of 144 globin chains and 36 linkers. Four different types of globin chain (*a*, *b*, *c* and *d*) unite to form *abcd* tetramers in which the *a* subunit is covalently linked to *b* and *c* by disulphide bridges. Three such tetramers form the dodecameric head of the one-twelfth protomers which are further stabilized by the presence of one copy of each of the three linker chains *L*1, *L*2 and *L*3. The latter protrude from the head into the centre of the particle giving the protomer a mushroom-like appearance and are primarily responsible for stabilizing the structure of the full particle. It is also worth noting that only quite recently the primary structures of the HbLt linker chains became available and a fourth linker, *L*4, was also reported (Kao *et al.*, 2006 ▶).

From a physico-chemical viewpoint, besides the HbLt, one of the erythrocruorins which has been most extensively studied is HbGp, an oligochaete earthworm readily encountered in the south-east of Brazil and whose molecular mass was originally reported to be of the order of 3.1 MDa (Costa *et al.*, 1988 ▶). More recently this value has been questioned after a careful re-examination using analytical ultracentrifugation consistently yielded values between 3.6 and 3.7 MDa (Carvalho *et al.*, 2009 ▶). This value is compatible with those described above for HbLt and with the fact that at alkaline pH HbGp dissociates into monomeric and disulphide-linked trimeric globin chains as well as non-globin linkers (Imasato *et al.*, 1995 ▶). More specifically, the monomeric HbGp globin chain (*d*) has been fully sequenced and presents 57% amino acid sequence identity with its homologue HbLt, indicating a common evolutionary origin (Bosch Cabral *et al.*, 2002 ▶). All these data suggest that HbGp is expected to present an overall architecture very similar to that seen for HbLt. This is borne out by results from SAXS studies (Gelamo *et al.*, 2004 ▶; Krebs *et al.*, 2004 ▶) as well as from mass spectrometric measurements (Oliveira *et al.*, 2007 ▶; Martin *et al.*, 1996 ▶) which have shown a great deal of similarity between the two haemoglobins. Furthermore, the stability of the HbGp particle as a function of pH, temperature and the presence of different detergents, as well as the close interdependence of subunit dissociation and auto-oxidation of the haem groups, have all been either well characterized or work is in progress to achieve this goal, making it, together with HbLt, one of the best studied members of the family described to date (Santiago *et al.*, 2007 ▶, 2008 ▶; Oliveira *et al.*, 2008 ▶). The relevance of these physico-chemical studies is also due to the fact that, since HbGp is an extracellular haemoglobin freely circulating in the worm haemolymph, besides its main oxygenation function it could possibly perform several other unknown biological roles as a carrier of small-molecular-weight biomolecules.

In spite of the significant advances made in understanding the architecture of these giant molecular assemblies, many outstanding questions remain concerning the details of the molecular mechanisms involved in their complex physiology. Some of the current limitations are the consequence of the relatively low resolution of the structures thus far reported for the full particles, 3.5 Å and 6.2 Å for *L. terrestris* (Royer *et al.*, 2006 ▶) and *A. marina* (Royer *et al.*, 2007 ▶), respectively. However, these limitations have been partially overcome by higher resolution studies of the (*abcd*)_3_ dodecamer in isolation which is believed to form the allosteric core of the molecule (Strand *et al.*, 2004 ▶). Nevertheless, the interest in obtaining closer to atomic resolution structures for the full particle in different ligand-bound states would appear to strongly justify continued effort in the search for high-quality crystals of erythrocruorins from other species. The many previously described studies on HbGp, together with the research currently under way regarding its oligomeric stability, make it an attractive system on which to work and in this paper we describe progress made towards its structure determination.

## 2. Material and methods

The whole HbGp complex was purified directly from adult earthworms as previously described (Agustinho *et al.*, 1996 ▶, 1998 ▶). Preparation of cyanomet-HbGp was made by addition of five-fold excess, relative to haem, of potassium ferrocyanide and potassium cyanide. After incubation for 1 h, a dialysis against the original buffer was performed to eliminate excess oxidation reagents (Agustinho *et al.*, 1996 ▶, 1998 ▶; Carvalho *et al.*, 2009 ▶). Initial crystallization conditions were screened using the sitting-drop vapour-diffusion method employing the Nextal Classic and PEG suites. Crystals appeared in droplets composed of 0.5 µl of an 18 mg ml^−1^ solution of cyanomet-HbGp together with an equal volume of the reservoir solution (Classic Suit Screen conditions Nos. 44 and 63) equilibrated against 100 µl of the latter. After optimization, hanging drops were mounted under the following two crystallization conditions: (i) 1.2 *M* sodium citrate, 2.5 m*M* CaCl_2_, 50 m*M* Tris-HCl pH 7.5, and (ii) 10% PEG8000, 2.5 m*M* CaCl_2_, 50 m*M* Tris-HCl pH 7.5, where crystals of HbGp grew in 72 h. Crystals obtained under the condition containing sodium citrate also grew in the presence of 5% ethylene glycol or 5% glycerol. The cryo-protection procedure consisted of the addiction of small volumes (0.5 µl) of cryo-protective solution to the droplet containing the crystals up to a final concentration of 10% ethylene glycol. Crystals were picked from the droplets and flash frozen in liquid nitrogen. X-ray images were collected at the same temperature with an ADSC Quantum 315 detector using synchrotron radiation of wavelength 1.00 Å at beamline X29A of the NSLS (Brookhaven National Laboratory, USA). The crystal-to-detector distance was set to 350 mm and oscillation images were collected at intervals of 0.5° with an exposure time of 1 s. Diffraction data were processed using *iMOSFLM* (Leslie, 2006 ▶) and *SCALA* from the CCP4 package (Collaborative Computational Project, Number 4, 1994 ▶; Potterton *et al.*, 2004 ▶). The phase problem was solved by molecular replacement employing the one-twelfth protomer of the erythrocruorin from *L. terrestris* as the search model (Protein Data Bank code 2gtl) using the program *Phaser* (McCoy *et al.*, 2007 ▶). The haem groups were omitted during this procedure.

## 3. Results and discussion

**Figure 1:**
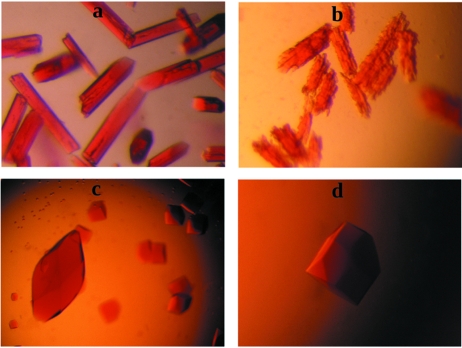
Crystals of HbGp obtained under two different conditions. (*a*) 10% PEG8000, 2.5 m*M* CaCl_2_, 50 m*M* Tris-HCl pH 7.5, which grew to approximate maximum dimensions of 0.1 mm. These crystals readily deteriorate after about a week (*b*) and often disappear altogether. Crystals obtained in the presence of 1.2 *M* sodium citrate, 2.5 m*M* CaCl_2_, 50 m*M*, Tris-HCl pH 7.5, can grow to a maximum dimension of almost 1 mm (*c*), but the best diffraction patterns were obtained from well formed crystals with typical dimensions of approximately 0.1 mm in all directions (*d*).

HbGp was successfully purified to homogeneity and crystals of the cyanomet derivative obtained under two different conditions, both at pH 7.5 and in the presence of calcium, as described above. The condition containing PEG8000 was not pursued further as these crystals proved difficult to reproduce, were very fragile during manipulation and usually grew as clusters which degraded rapidly with time, normally dis­appearing after a few days [Figs. 1(*a*) and 1(*b*) ▶]. No significant diffraction pattern could be collected from these crystals. On the other hand, those obtained from the condition containing sodium citrate were easy to reproduce, stable, well formed and resistant to manipulation (Fig. 1*c* ▶). They also provided significant initial diffraction patterns which extended to approximately 4.8 Å resolution on a rotating-anode generator (data not shown). However, these crystals were highly sensitive during transfer to the cryo-protectant solution. This problem could be significantly diminished by growing the crystals in the presence of 5% ethylene glycol followed by successively adding small volumes of a more concentrated ethylene glycol solution up to a final concentration of 10%. This procedure minimized the osmotic shock, and typically resulted in several unbroken crystals in the droplet suitable for flash cooling in liquid nitrogen and subsequent diffraction. The same procedure was adopted when using glycerol as cryo-protectant but none of the crystals obtained under these conditions provided useful diffraction data. Larger crystals, of approximately 0.5 mm in length, generally did not withstand the cryogenic cooling and the data described here were therefore collected from smaller or medium-sized crystals (Fig. 1*d* ▶).

**Figure 2:**
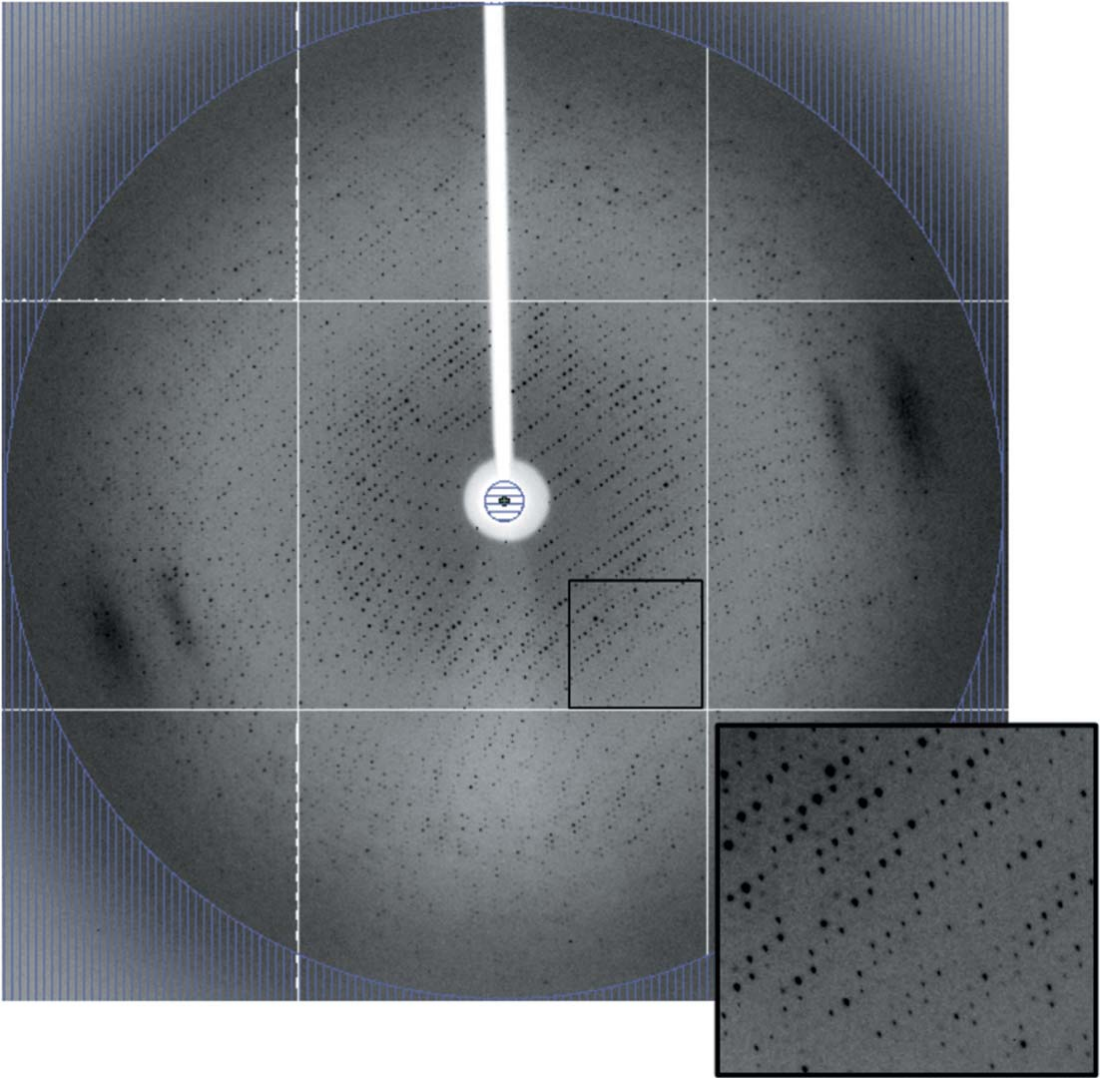
A typical diffraction image from a cyanomet HbGp crystal. The resolution at the edge of the detector is 3.10 Å and the oscillation angle is 0.5°. The insert refers to the highlighted region.

**Figure 3:**
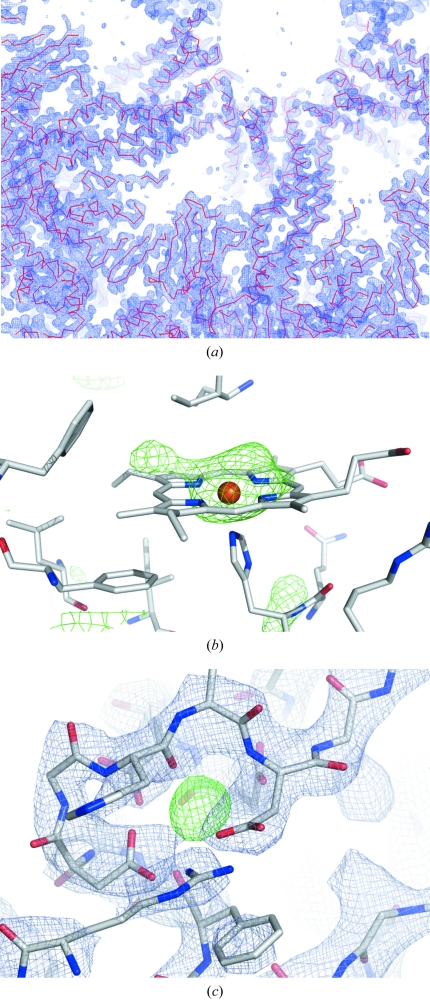
Electron density maps showing (*a*) an overview of part of the 2*F*_o_ − *F*_c_ map of the full particle contoured at 2σ, (*b*) an *F*_o_ − *F*_c_ omit map of the region of one of the haem groups contoured at 3σ, and (*c*) 2*F*_o_ − *F*_c_ (at 2σ, blue) and *F*_o_ − *F*_c_ (at 4σ, green) maps of the region of a predicted calcium binding site.

Diffraction data were obtained from the cyanomet derivative of HbGp and diffracted to a minimum *d*-spacing of 3.2 Å. Crystal parameters and diffraction data statistics are summarized in Table 1 ▶ and a typical diffraction image is shown in Fig. 2 ▶. The space group was initially determined to be *I*222 or *I*2_1_2_1_2_1_, with unit-cell parameters of *a* = 272.68, *b* = 319.90 and *c* = 333.18 Å. From the total of 1105247 measured reflections, 237062 independent reflections were obtained with an *R*_merge_ of 11.2% (50.0% in the outermost shell). The data set was 99.8% (99.2%) complete at a final resolution limit of 3.2 Å. The phase problem was readily solved and the space group ambiguity broken by molecular replacement using the crystallographic structure of the homologue from *L. terrestris* as template (2gtl). The search model corresponded to a one-twelfth protomer from which the haem groups had been excluded in order to be used subsequently as a criterion for evaluating the molecular replacement solution. The localization of the first protomer yielded an *R* value of 57.2% and a log likelihood gain of 1446. These values improved to 53.5% and 5509, respectively, after localization of the second protomer and then to 49.6% and 12050 after the third. The initial electron density maps clearly showed evidence of haem groups at the expected positions as well as many side chains including disulphide bonds. Furthermore, densities consistent with possible calcium ions are observed close to the linker chains in positions equivalent to those seen for HbLt. The overall electron density is consistent with a similar subunit composition as that seen in HbLt. Fig. 3 ▶ shows some examples of electron density maps from selected regions.

**Figure 4:**
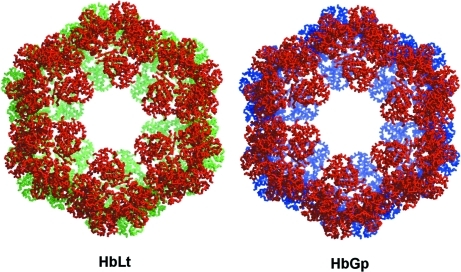
The reconstructed HbGp globin chains based on the molecular replacement solution using a protomer of HbLt as the search model. The structure shows the offset of the vertices of the upper disc with respect to the lower one, characteristic of the type I architecture. This is most evident towards the centre of the structure where subunits from the two layers can be clearly visualized. HbLt is shown for comparison. In both images red refers to subunits of the upper disc whilst green (or blue) refer to those of the lower disc.

Three mushroom-like protomers comprise the asymmetric unit. This corresponds to one-quarter of the full particle, consisting of 36 globin chains and nine linkers. The full particle lies on a special position at the intersection of the three twofold axes with two of the three protomers of the asymmetric unit belonging to one hexagonal disc and the third to the other. The full particle (Fig. 4 ▶) is therefore generated by the application of crystallographic symmetry and clearly shows the vertices of the six protomers in one layer to be staggered with respect to those in the other, identifying it to be of the type I architecture, very similar to that observed for HbLt (Royer *et al.*, 2007 ▶).

The content of the asymmetric unit of the crystals described here is considerably smaller than that for HbAc and HbLt which have one and two full particles in the asymmetric unit, respectively. This may represent an advantage in the search for higher resolution data and it is already encouraging that those reported here are already the highest described to date. This has the potential to be further extended if crystal optimization together with the use of appropriate synchrotron sources can be successfully employed. This may open up the route towards higher resolution studies of the full particle bound to different ligands and therefore a better understanding of cooperativity and allosterism. Moreover, studies on the binding of small biomolecules to HbGp would potentially contribute to the deeper understanding of some unknown roles of the linker chains besides their assumed requirement to maintain the oligomeric structure of the complex.

Currently the refinement of the structure is hampered by a lack of complete amino acid sequences for six (or seven) of the expected seven (or eight) chains. We are in the process of addressing this using a combination of mass spectrometry and recombinant DNA technologies.

## Figures and Tables

**Table 1:** Crystal and data collection statistics

Space group	*I*222
Cell dimensions *a*, *b*, *c* (Å)	272.68, 319.90, 333.18
Detector	ADSC Quantum 315
X-ray source	NSLS X29c
Wavelength (Å)	1.00
Resolution range (Å)	50.0–3.2 (3.37–3.20)
Redundancy	4.7 (4.2)
*R*_merge_ (%)†	11.2 (50.0)
Completeness (%)	99.8 (99.2)
Total reflections	1105247 (145116)
Unique reflections	237062 (34189)
Mean *I*/σ*I*	11.2 (3.1)
*B*-factor in Wilson plot (Å^2^)	60.4
Asymmetric unit contents	Three protomers
Molecular replacement statistics (log likelihood gain/*R* value‡)	12051/49.6%

† *R*_merge_ = Σ_*hkl*_Σ_*i*_|*I*_*i*_(*hkl*) − 〈*I*(*hkl*)〉|/Σ_*hkl*_Σ_*i*_ *I*_*i*_(*hkl*), where *I*_*i*_(*hkl*) is the observed intensity of the measured reflection and 〈*I*(*hkl*)〉 is the averaged intensity over equivalent reflections from different measurements.

‡ *R* is the conventional crystallographic *R*-factor, Σ||*F*_obs_| − |*F*_calc_||/Σ|*F*_obs_|, where *F*_obs_ and *F*_calc_ are the observed and calculated structure factors, respectively.
